# Influence of Indomethacin on Steroid Metabolism: Endocrine Disruption and Confounding Effects in Urinary Steroid Profiling of Anti-Doping Analyses

**DOI:** 10.3390/metabo10110463

**Published:** 2020-11-14

**Authors:** Anna Stoll, Michele Iannone, Giuseppina De Gregorio, Francesco Molaioni, Xavier de la Torre, Francesco Botrè, Maria Kristina Parr

**Affiliations:** 1 Institute of Pharmacy (Pharmaceutical and Medical Chemistry), Freie Universität Berlin, 14195 Berlin, Germany; anna.stoll@fu-berlin.de; 2 Laboratorio Antidoping Federazione Medico Sportiva Italiana, 00197 Rome, Italy; micheleiannone14@gmail.com (M.I.); degregorio.giuseppina@gmail.com (G.D.G.); molaioni@gmail.com (F.M.); xavier.delatorre@gmail.com (X.d.l.T.); Francesco.Botre@unil.ch (F.B.); 3 Synathlon—Quartier Centre, ISSUL—Institut des Sciences du Sport, Université de Lausanne, 1015 Lausanne, Switzerland

**Keywords:** NSAID, inhibition, doping, aldo-keto reductases, endocrine disruption

## Abstract

Anabolic androgenic steroids (AAS) are prohibited as doping substances in sports by the World Anti-Doping Agency. Concentrations and concentration ratios of endogenous AAS (steroid profile markers) in urine samples collected from athletes are used to detect their administration. Certain (non-prohibited) drugs have been shown to influence the steroid profile and thereby sophisticate anti-doping analysis. It was shown in vitro that the non-steroidal anti-inflammatory drug (NSAID) indomethacin inhibits selected steroid-biotransformations catalyzed by the aldo-keto reductase (AKR) 1C3, which plays a key role in the endogenous steroid metabolism. Kinetic parameters for the indomethacin-mediated inhibition of the AKR1C3 catalyzed reduction in etiocholanolone were determined in vitro using two comparing methods. As NSAIDs are very frequently used (not only) by athletes, the inhibitory impact of indomethacin intake on the steroid metabolism was evaluated, and steroid profile alterations were detected in vivo (one male and one female volunteer). Significant differences between samples collected before, during or after the intake of indomethacin for selected steroid profile markers were observed. The presented results are of relevance for the interpretation of results from doping control analysis. Additionally, the administration of NSAIDs should be carefully reconsidered due to their potential as endocrine disruptors.

## 1. Introduction

**Figure 1:**
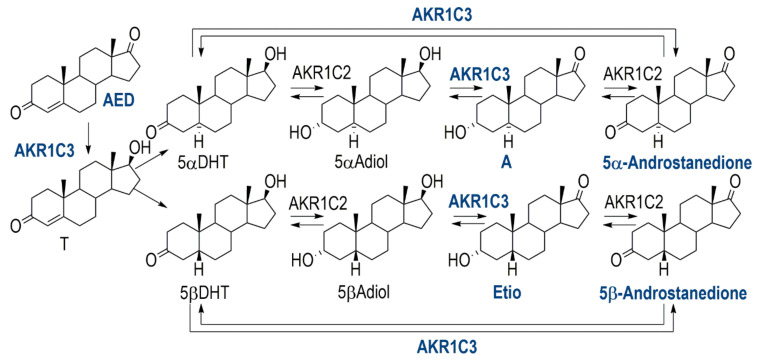
Metabolism of endogenous anabolic androgenic steroids (EAAS); blue and bold: substrates and enzymes used in this publication.

Anabolic androgenic steroids (AAS) are very frequently used drugs in sports [1]. Their use as doping agents is prohibited in and out of competition by the World Anti-Doping Agency (WADA; class S1 in the WADA prohibited list) [2]. The analytical detection is challenging, especially if so-called pseudo endogenous AAS (e.g., exogenous testosterone) are used as performance enhancing substances, due to their high similarity to the naturally occurring endogenous AAS (EAAS). To detect the misuse of those pseudo endogenous and some synthetic AAS, anti-doping laboratories monitor in a first step, concentrations and concentration ratios of selected EAAS according to the WADA technical document TD2018EAAS in urine samples collected from the athletes [3]. In case of misuse of pseudo endogenous AAS or some synthetic AAS, those steroid profile markers are altered and a confirmative method using gas chromatography combustion isotope-ratio mass-spectrometry (GC-c-IRMS) is applied. Since it has been shown that ratios of urinary steroids are stable over months and even years in adult humans [4,5,6] but show interindividual variations, the steroidal module of the Athlete Biological Passport (ABP) was introduced by WADA in 2014 [7]. With this longitudinal monitoring model, it is possible to better detect intraindividual changes, and hence the potential misuse of AAS. However, it was shown that besides various endogenous and exogenous parameters, the intake of selected (non-prohibited) drugs can influence the individual steroid profile and lead to suspicious testing results [8,9,10]. To better understand how changes in the steroid profile can occur after the intake of specific drugs, it is helpful to understand and further investigate the steroid metabolism and potential points of interference. One enzyme-family which plays a key role in the metabolism of EAAS are aldo-keto-reductases (AKR; Figure 1). In this study we focused on the AKR1C3, which is known to oxidize 17-hydroxy steroids to their corresponding 17-oxo metabolites and vice versa. It was reported that the reduction route is favored in vivo [11]. Furthermore, it was reported by Byrns et al. that the non-steroidal anti-inflammatory drug (NSAID) indomethacin inhibits the AKR1C3 catalyzed reduction in androst-4-ene-3,17-dione in vitro selectively over the closely related AKR1C2 and AKR1C1 [12,13]. No further investigations have been made on the inhibitory effect of indomethacin on 5β-androstanes metabolized by AKR1C3. As NSAIDs are very frequently used drugs (not only) among athletes [14,15], this work aims to further investigate the influence of indomethacin on the steroid metabolism in vitro and wants to show the relevance of indomethacin on the urinary steroid profile in vivo. Hence, the work consists of an in vitro part and an in vivo application trial. The in vitro experiments were analyzed spectro-fluorometrically in real time and by gas chromatography coupled to a quadrupole-time-of-flight mass spectrometer (GC-QToF) as confirmative tests. For the in vivo part, indomethacin was administered to one male and one female volunteer in therapeutic doses over 14 days. Urine samples before, during and after the administration were collected and analyzed. The results first give ideas on the impact of indomethacin intake on steroid profiles in doping control analysis and its potential mechanism of endocrine disruption.

## 2. Results

### 2.1. Qualitative Incubation In Vitro

**Figure 2:**
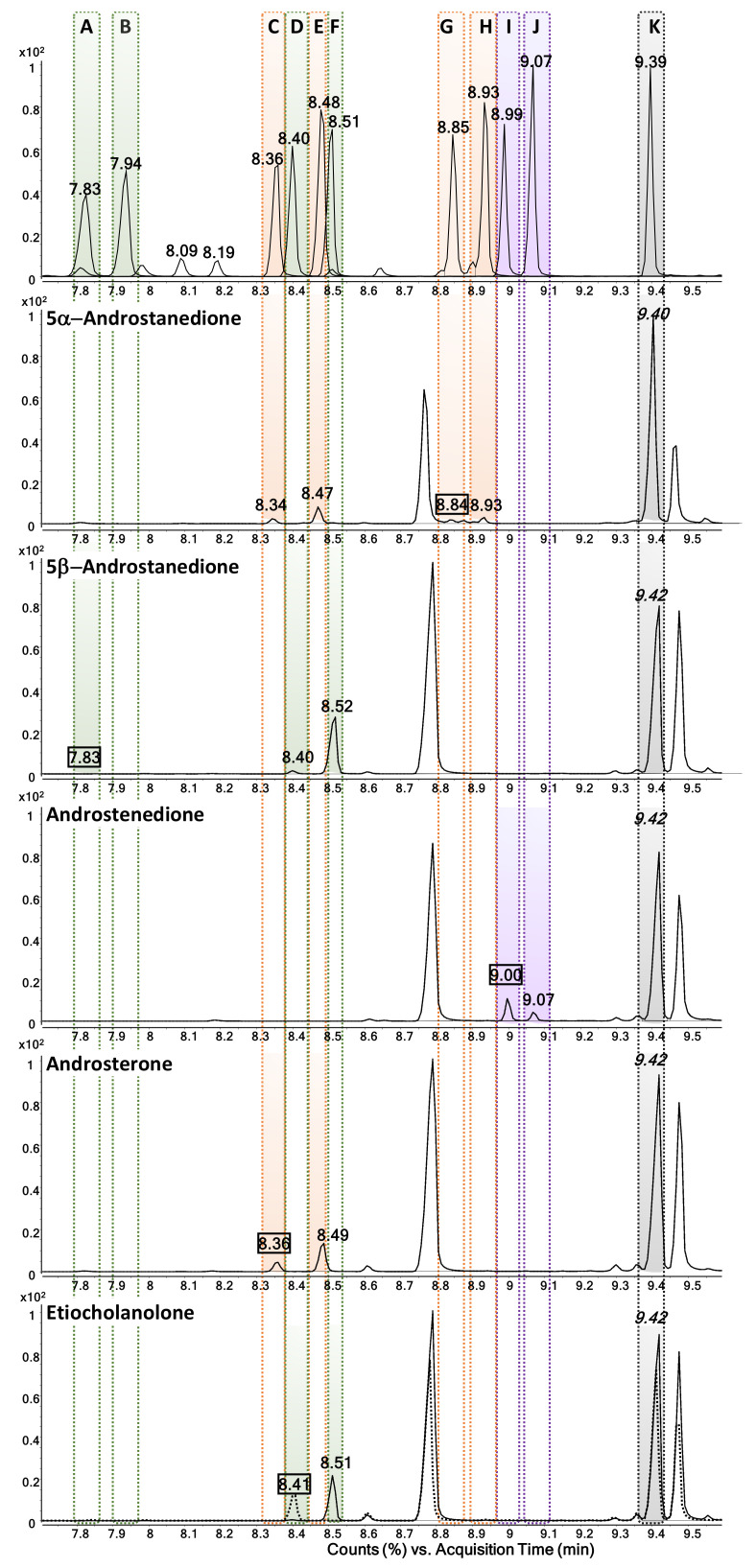
Chromatogram of EAAS standards (upper chromatogram) and steroids detected in overview-incubations (chromatograms below) analyzed on GC-MS with substrates of incubation indicated in upper left corner of each chromatogram and highlighted by framed retention time (RT); colored peaks corresponding to: A: 5βAD (RT: 7.83, 8.09 min, derivatization isomers); B: 5βDHT (RT: 7.94; 8.19 min, derivatization isomers); C: And (RT: 8.36 min); D: Etio (RT: 8.40 min); E: 5αAdiol (RT: 8.48 min); F: 5βAdiol (RT: 8.51 min); G: 5αAD (RT: 8.85 min); H: 5αDHT (RT: 8.93 min); I: AED (RT: 8.99 min); J: T (RT: 9.07 min); K: MeT (RT: 9.39 min; internal standard); orange peaks indicate 5α-androstanes, green peaks indicate 5β-androstanes, violet peaks correspond to T and AED, MeT is colored in black. Peaks not indicated with RT do not correspond to any EAAS standards.

With the applied GC-MS (gas chromatography-mass spectrometry) method, all EAAS of interest were sufficiently separated (no interference between steroids was expected to occur simultaneously during the different incubations). All analyzed EAAS standards are depicted in the upper chromatogram in Figure 2. For all background incubations (absence of enzyme) no other steroids besides the substrate were detectable. The internal standard (17α-methyltestosterone; substance K in Figure 2) was detected in all samples.

Figure 2 shows chromatograms of samples after enzymatic incubations in solid lines. As no substrate was detected after the incubation of Etio (etiocholanolone, substance D in Figure 2) the sample chromatogram (solid line) was superimposed by the chromatogram of the background-incubation (dotted line). A detailed display of chromatograms of all performed incubations and background samples is available as supplementary data (Supplement S1). Chromatograms are displayed as total ion current chromatograms. Hence, peaks originating from the incubation media are also present. This is assumed to be the case for the two big peaks at 8.79 min and at 9.47 min, as both are also present in background-samples (without enzyme). They are hence neglected in the results presentation and discussion.

In the following paragraph, detailed outcomes of individual steroid incubations with AKR1C3 (aldo-keto reductase 1C3) will be described. After incubation of 5αAD (5α-androstanedione, substance G in Figure 2), small amounts of substrate were detected. In addition, minor amounts of 5αDHT (5α-dihydrotestosterone, substance H in Figure 2) and larger amounts of And (androsterone, substance C in Figure 2) and 5αAdiol (5α-androstane-3α,17β-diol, substance E in Figure 2) were detected. All of these compounds have a 5α-androstane structure in common and are highlighted in orange in Figure 2.

After incubation of 5βAD (5β-androstanedione, substance A in Figure 2) no or small amounts of substrate, but peaks corresponding to 5βAdiol (5β-androstane-3α,17β-diol, substance F in Figure 2) and Etio (substance D in Figure 2), were detected. Furthermore, very minor amounts of 5βDHT (5β-dihydrotestosterone, substance B in Figure 2) were detectable in one of two replicates (not visible in Figure 2). All of these compounds have a 5β-androstane structure in common and are highlighted in green in Figure 2.

After incubation of AED (androst-4-ene-3,17-dione, substance I in Figure 2) with AKR1C3, AED itself and its metabolite T (testosterone, substance J in Figure 2) were detected. Both compounds are highlighted in violet in Figure 2.

After incubation of And (substance C in Figure 2) with AKR1C3, peaks corresponding to And and 5αAdiol (substance E in Figure 2) were detected. Only minor amounts of the substrate were detected (small peak at 8.36 min with framed retention time (RT) in androsterone chromatogram in Figure 2). And and 5αAdiol share a 5α-androstane structure and are hence highlighted in orange in Figure 2.

After incubation of Etio (substance D in Figure 2) the substrate itself was detected in only one of the two replicates (indicated as dotted peak at 8.41 min corresponding to Etio detected in the background sample). The metabolite 5βAdiol (substance F in Figure 2) was present in both replicates. As Etio and 5βAdiol share a 5β-androstane structure they are highlighted in green in Figure 2.

### 2.2. K_m_ Determination In Vitro

**Figure 3:**
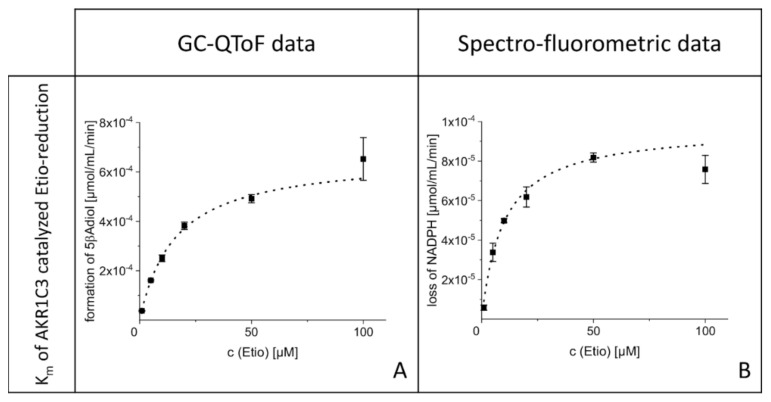
Michaelis-Menten curve of Etio with AKR1C3 generated with data from GC-QToF data (**A**): K_m_ = 15.8 µM (SE = 0.9 µM) and spectro-fluorometric measurement (**B**): K_m_ = 9.7 µM (SE = 1.4 µM).

Experiments to determine the Michaelis-Menten constant (K_m_) of AKR1C3 catalyzed Etio metabolism were carried out in 96-well plates to detect real-time changes in fluorescence intensities. The K_m_ determined with the spectro-fluorometric method was 9.7 µM (standard error of the mean; SE = 1.4 µM; Figure 3B). As previously described, analysis of terminated incubations was additionally carried out using a GC-QToF instrument. The determined K_m_ with this method was 15.8 µM (SE = 0.9 µM; Figure 3A). Michaelis-Menten curves of both measurements are depicted in Figure 3.

### 2.3. IC_50_ Determination In Vitro

For the measurements performed with the spectro-fluorometric method, the half maximal inhibitory constant (IC_50_) could not be calculated. Several background samples (enzyme substituted by phosphate buffered saline (PBS) 0.1 M) showed higher initial velocities than the samples which contained enzyme. Hence, a subtraction of background-velocities from initial velocities of samples would have resulted in negative values and hence negative enzyme activities would have been determined. Therefore, IC_50_ values were determined with the data obtained by GC-QToF analysis.

**Figure 4:**
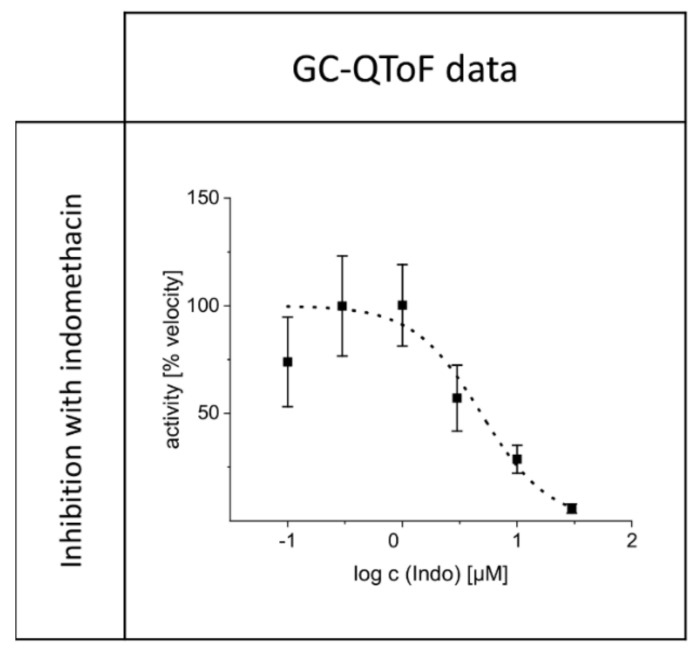
Inhibition curve generated from GC-QToF data: IC_50_ = 4.8 µM (SE = 1.0 µM).

The IC_50_ determined using the mass-spectrometric method was successful and resulted in a value of 4.8 µM (SE = 1.0 µM). The corresponding curve is depicted in Figure 4.

### 2.4. In Vivo Administration Trial

**Figure 5:**
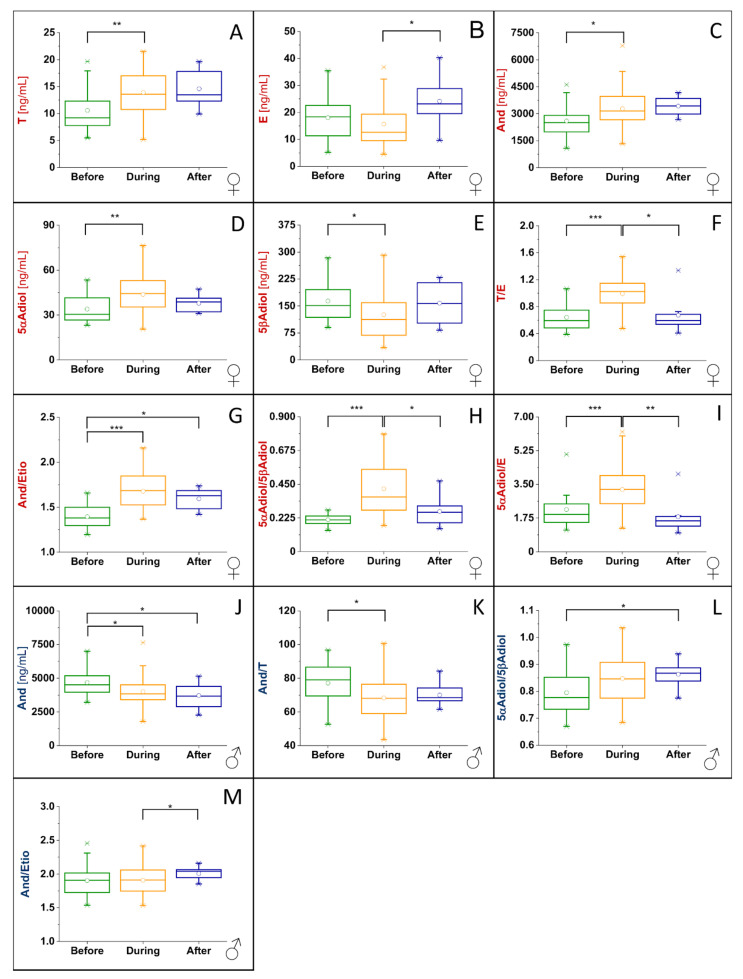
Boxplots of steroid profile markers with significant differences between at least two groups. (**A**–**I**): female volunteer; (**J**–**M**): male volunteer; * significant difference with *p* < 0.0167; ** very significant difference with *p* < 0.0033; *** highly significant difference with *p* < 0.00033.

To illustrate all significant changes in the steroid profile markers of the male and the female volunteers, boxplots of steroid profile markers where significant differences were found between at least two groups are displayed in Figure 5.

In the male volunteer, significant changes between at least two groups, i.e., before and during administration, were detected for And (Figure 5J), And/T ratio (Figure 5K), And/Etio ratio (Figure 5M) and 5αAdiol/5βAdiol ratio (Figure 5L). The concentration of And decreased by 15% (difference in median) during the administration of indomethacin compared to the concentration measured before the administration and decreased by 21% (difference in mean) after the administration compared to before the administration. The And/T ratio decreased by 11% during the administration compared to the ratios before (difference in mean values). The And/Etio ratio increased by 5% (difference in mean) after the administration compared to during administration and the 5αAdiol/5βAdiol ratio increased by 9% (difference in mean) after administration compared to before administration. In the female volunteer, significant differences were detectable for all examined steroid profile markers except for Etio concentration and And/T ratio. Changes solely between the groups before and during administration of indomethacin were detectable for T (Figure 5A), And (Figure 5C), 5αAdiol (Figure 5D) and 5βAdiol (Figure 5E). Changes solely between the groups during and post administration were detectable for E (epitestosterone, Figure 5B). Changes between the groups before/during and during/after were detectable for T/E (Figure 5F), 5αAdiol/5βAdiol (Figure 5H) and 5αAdiol/E (Figure 5I) ratios. Changes between the groups before/during and before/after were significant for And/Etio ratio (Figure 5G).

**Figure 6:**
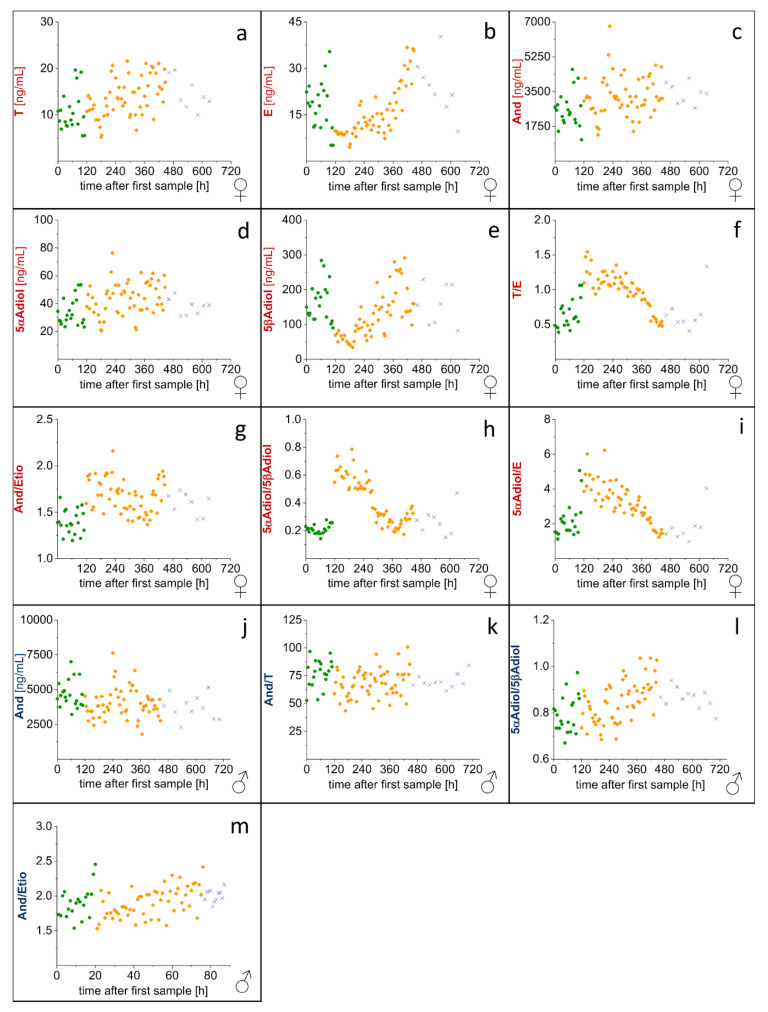
Time course of steroid profile markers which changed significantly over time; (**a**–**i**): female volunteer; (**j**–**m**): male volunteer.

Furthermore, concentrations and concentration ratios of steroid profile markers, which showed significant changes between at least two groups, were plotted over the whole sampling time. The first sampling point was at t = 0 h. All curves are displayed in Figure 6. From this visualization it is observable that in particular, the concentration of E and 5βAdiol and the T/E, 5αAdiol/E and 5αAdiol/5βAdiol ratios in the female volunteer change a lot over time (Figure 6b,e,f,h and i). Additionally, for the And/Etio ratio in the female volunteer (Figure 6g) and for the 5αAdiol/5βAdiol ratio in the male volunteer (Figure 6l) an increase during the administration of indomethacin is clearly visible.

**Figure 7:**
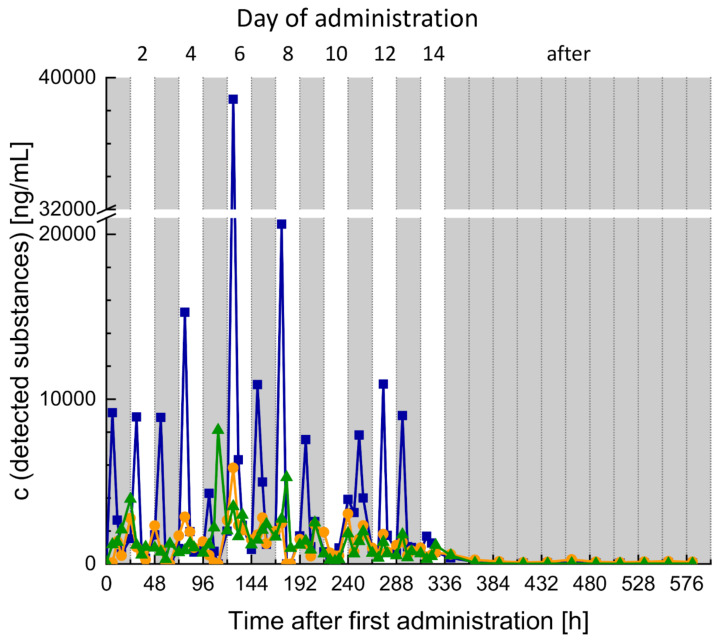
Measured concentrations of indomethacin (blue squares) and its metabolites O-desmethoxyindomethacin (orange circles) and *N*-deschlorobenzoylindomethacin (green triangles) in urine samples collected from the male volunteer. Sampling points for one day were: 7 h, 13 h, 18 h and 22 h.

Indomethacin and its two evaluated metabolites were detected over the whole time of administration. Thereby, the highest concentration of indomethacin was determined in the samples collected after the administration of the drug (13 h). The two following samples before the next intake (18 h and 22 h) were much lower than the detected peak-concentration. Samples collected after the administration period (day 15 and later) contained, if at all, only trace amounts of indomethacin or its examined metabolites (Figure 7).

## 3. Discussion

### 3.1. In Vitro Overview Incubation

Overview incubations were performed to examine the suitability of different endogenous steroids as substrate for the subsequent inhibition experiments. Important requirements were:

- The substrate is an endogenous steroid, which plays a role in the steroid module of the Athlete Biological Passport of the World Anti-Doping Agency;
- No side reaction is taking place besides the formation of the desired product;
- The initial velocity is fast enough to be monitored spectro-fluorometrically.

From those requirements it was assumed that a substrate which shows nearly complete formation of product, with no formation of side products after 5.5 h would be an adequate candidate for the following experiments. The requirement of no side reactions guarantees that a “one-to-one reaction” is taking place. Therefore, NADPH (β-Nicotinamide-adenine-dinucleotide-phosphate) consumption measured spectro-fluorometrically directly correlates with product formation measured on the GC-QToF, and the data from spectro-fluorometric measurements and chromatographic confirmation are comparable. The nearly complete formation of the product after a (rather long) incubation period of 5.5 h indicates a higher reaction velocity which would result in a higher sensitivity of the spectro-fluorometric analysis.

For all enzymatic incubations, the formation of product(s) was observed. Hence, a negative effect of methanol or acetonitrile (used as solvents for steroid substrates) on the outcome of the qualitative experiments was not expected.

Five endogenous steroids were chosen for preliminary experiments. 5α- and 5β-androstanedione formed three products each, which was not expected, since all other examined EAAS only showed the formation of one product. Following the pattern of all other observed products, we assumed that also 5α- and 5β-androstanedione would form their 17-hydroxy-metabolite, 5α- or 5β-dihydrotestosterone, respectively. However, we only detected small amounts, of the latter and of the substrates, if any. Instead, we observed the formation of And or Etio and 5αAdiol or 5βAdiol, respectively. Those findings are in accordance with the literature, where AKR1C3 is described to act as 3α-hydroxysteroid dehydrogenase (3αHSD) as well as 17βHSD [16]. Further investigations would be interesting to evaluate the complex kinetics and the metabolite formation sequence to gain further insights into enzyme characteristics.

In the analyzed samples of AED, And and Etio, only one product was detected. Whereas AED and And formed their corresponding product T and 5αAdiol in relatively small amounts in both replicates, Etio was converted nearly completely into its product 5βAdiol in one of two replicates (Figure 2). Due to the complete metabolization and the absence of any side reactions, Etio was chosen as the substrate for the subsequent in vitro kinetic experiments.

### 3.2. Kinetic Measurements In Vitro

To prevent the potential interfering effects of acetonitrile or methanol (used as solvent of the steroid substrate for qualitative incubations) with the enzyme, Etio was dissolved in DMSO (dimethyl sulfoxide) for all the kinetic measurements. Constant volumes of DMSO and ethanol were added to all incubations (with and without indomethacin and to samples without enzyme) to generate comparable results.

To find the optimal substrate concentration for the subsequent inhibition experiments, the K_m_ value of the AKR1C3-catalyzed reduction in Etio is needed. These values were determined experimentally since, to our knowledge, no kinetic data on the desired reaction are available in the literature. The K_m_ values from the spectro-fluorometric and mass spectrometric measurements (K_m_(fluoro) = 9.7 µM, standard error of the mean; SE = 1.4 µM and K_m_(QToF-MS) = 15.8 µM (SE = 0.9 µM) show that the method used to analyze the kinetic experiments has an impact on the determined K_m_ value. Despite the difference in the K_m_ values determined with the two methods, the examined reaction has an efficiency comparable to other steroid-reductions catalyzed by AKR1C3 reported in the literature [12,16]. The reactions of steroid substrates are relatively inefficient compared to other substrates converted by AKR1C3, e.g., 9,10-phenanthrenequinone (K_m_ = 1.5 ± 0.7 µM) [12,17]. This may explain why the spectro-fluorometric method showed good results for the K_m_ determination, but not for inhibition experiments. Initial velocities in the inhibition experiments were generally lower than those determined in the K_m_ experiments, resulting in lower sensitivities. The presence of indomethacin may be another explanation for the unsuitability of the spectro-fluorometric method for the inhibition experiment. In background reactions, we observed that large amounts of indomethacin led to quenching of the fluorescence intensity generated by NADPH. Generally, subtracting the background signal from the sample-signal should compensate for this difference. Nevertheless, we observed that in particular, background samples with high amounts of indomethacin resulted in higher initial velocities than the corresponding samples with the enzyme. As the evaluation of those data would result in negative enzyme activities and in activities far above 100%, the applied spectro-fluorometric method is not suitable to determine the IC_50_ value of the inhibition of AKR1C3 catalyzed reduction in Etio. Due to those difficulties in the spectro-fluorometric method, the IC_50_ was determined using mass spectrometry. The determined value of 4.8 µM (SE = 1.0 µM) was transformed into an inhibition constant (K_I_) using the “IC_50_-to-K_I_-converter” [18]. Byrns et al. reported a competitive inhibition type of indomethacin on the reduction in androstenedione by AKR1C3 [12]. Assuming that the inhibition of the AKR1C3 catalyzed reduction in Etio follows the same pattern, the calculated K_I_ would be 2.2 µM. This result is in accordance with findings from the literature, where different substrates were used [12,19,20]. The determined K_I_ lies on the upper range of the therapeutic blood-plasma/serum concentration of the drug (0.84–2.8 µM) [21], resulting in the hypothesis for the in vivo experiments that therapeutic doses of indomethacin may lead to relevant inhibition of AKR1C3.

### 3.3. In Vivo Administration Trial

As AKR1C3 plays a crucial role in the metabolism of EAAS, used as markers in doping control analysis, we assumed that the intake of therapeutic doses of indomethacin may lead to changes in the steroidal profile. Since the aim of this study was getting a first idea on the detectability of changes in the steroid profile after the intake of therapeutic doses of indomethacin over 14 days, only one male and one female volunteer were included in the study. Despite this limited number of volunteers, we are convinced that the presented results are relevant for the anti-doping community and other fields where steroid quantification is of importance. As the knowledge of so-called confounding factors is of paramount importance for the interpretation of steroid profiles [10], this study will be of value for improved interpretation of steroid profiles. To enhance the exploration of factors influencing the steroid profile, we want to share our findings to raise awareness and induce further in-depth research.

Statistical analysis of in vivo data showed significant changes between the groups (before, during and after indomethacin administration) for several EAAS in both volunteers (Figure 5 and Figure 6). While in the male volunteer only four steroid profile markers changed significantly (A, And/T, And/Etio and 5αAdiol/5βAdiol), nine of the 11 markers changed in the female volunteer. This is in accordance with our previous study on the influence of ibuprofen intake on the steroid profile, where only a few steroid profile markers were affected in the male volunteer, while the majority of markers changed significantly in the female volunteer [22].

Due to the experimental setting, both individual and sexual differences may be explanations for this effect. However, sexual differences seem to be reasonable as an explanation. As females have lower concentrations of EAAS than males, their concentrations and concentration ratios monitored in the steroid profile of the Athlete Biological Passport are much more affected by exogenous and endogenous parameters (e.g., menstrual cycle, intake of contraceptives or emergency contraceptives, ethanol) [23,24]. Mullen et al. showed that the epitestosterone concentration changes significantly during the menstrual cycle, and hence the T/E and 5αAdiol/E ratios are significantly affected. Similar observations are true for the female volunteer in this study who did not take any oral contraceptives for at least six months before and during the study. In particular, the concentration of E, the T/E and the 5αAdiol/E ratios changed a lot over the whole collection time (Figure 6). The influence of physiological changes on these parameters may need further investigation. In a publication by Mareck-Engelke et al. [25] also considering the potential circadian rhythm and the menstrual cycle in females, the And/Etio ratio is reported to be the most stable, but also the 5αAdiol/5βAdiol ratios showed a coefficient of variation (CV) <30%. However, both ratios (A/Etio and 5αAdiol/5βAdiol) changed significantly in the female volunteer during indomethacin application. In addition, the concentrations of And and 5αAdiol increased significantly, while the concentration of 5βAdiol decreased significantly during the intake.

In the male volunteer, the picture is not as complex as in the female volunteer. The significant decrease in And/T ratio may be explained by the decrease in And during the intake of indomethacin. The 5αAdiol/5βAdiol ratio increased significantly during the intake of indomethacin, which is in accordance with the observations from the female volunteer.

For both volunteers, several steroid profile markers changed significantly under the influence of indomethacin but no criteria for a suspicious steroid profile during an anti-doping test were met for a sequence of successive samples. Nonetheless, selected concentrations and concentration ratios were significantly influenced by the intake of the drug. Additionally, the concentrations of indomethacin and its metabolites *O*-desmethylindomethacin and *N*-deschlorobenzoylindometacin were determined in the urine samples collected from the male volunteer to obtain some first information on the long-time influence of indomethacin on the steroid profile. It was found that the highest concentrations of indomethacin are detectable in the sample which was collected after the drug intake. The samples collected after this and before the next indomethacin administration contained much lower concentrations (Figure 7). This is in accordance with the half-life of 3–11 h [21]. Based on the half-life, and since indomethacin and its monitored metabolites are only detectable in urine samples collected close to the intake of the drug, one would assume that no differences in steroid profile markers are observable between samples collected before and after indomethacin intake. Nevertheless, there are a few significant changes in the steroid profile detectable between samples collected before and after the administration of indomethacin (Figure 5G,J,L). Other profile markers are affected during the intake of the drug but show no significant difference between the groups during and after indomethacin intake (Figure 5A,C–E,K). An explanation of these effects may be a prolonged influence of indomethacin on AKR1C3 and thus also the steroidal profile. To further investigate the significance for doping-control analysis and to reassess our hypothesis a study with a higher number of volunteers is already in preparation.

In addition to the impact on results interpretation in anti-doping analysis, the presented results are also relevant in evaluating potential endocrine disrupting substances (EDS). Like several other NSAIDs, indomethacin has anti-inflammatory, anti-pyretic and analgesic effects. It inhibits the cyclooxygenase and hence the formation of prostaglandins (PGs) which are responsible for the manifestation of pain, inflammation and fever. The literature reports that many putative EDSs inhibit the PG synthesis [26]. Additionally, the literature also discusses the role of PG synthesis inhibition by indomethacin in the mechanism of endocrine disruption [27,28]. However, it is accepted that probably not only the inhibition of PG synthesis is involved. Previous studies showed that indomethacin interferes with the testosterone production in various models [26,27,29,30,31,32]. Barkay et al. reported that plasma-testosterone levels in oligospermic men decreased significantly after an intake of indomethacin (75 mg over 90 days) [30]. In comparison, Knuth et al. reported an investigation with an administration of a lower dose and shorter duration (25 mg indomethacin per day over 14 days, i.e., similar to our administration). No significant change was detectable in the serum testosterone concentration in young, healthy male volunteers. This is in accordance with our presented findings, where no significant changes in the urinary testosterone concentration were detectable in the male volunteer. In the female volunteer, where steroid profile markers were showed to be more easily affected, significant changes in the urinary testosterone concentrations were detectable during the intake of indomethacin compared to pre-administration. Future investigations could hence investigate whether higher daily doses or prolonged administrations of indomethacin would also affect testosterone levels in the male volunteer. Additionally, studies on the impact of indomethacin on AKRs were published [12,19], and in vitro experiments presented in our study showed the effect of indomethacin on the AKR1C3 catalyzed reduction in Etio. All investigations commonly show that indomethacin inhibits AKR1C3 in vitro. Among other catalyzed reactions, this enzyme plays a key role in the metabolism of androgens, estrogens and progestagens [16]. As AKR1C3 is implicated in some hormone dependent malignancies and endocrine disorders, drugs selectively inhibiting AKR1C3 are investigated to be used as cancer treatment or drugs treating endocrine disorders for patients where this enzyme is overexpressed [33]. Our work demonstrates that the inhibition of AKR1C3 by indomethacin alters selected markers of the urinary steroid profile in healthy volunteers. In the context of AKR1C3 involvement in androgen, estrogen and progestin metabolism, an effect on estrogens may also be hypothesized. Based on our study and on findings from the literature, the risk potential of indomethacin and its role as endocrine disruptor should be reinvestigated.

## 4. Materials and Methods

### 4.1. Materials

Androst-4-ene-3,17-dione (AED), 17β-hydroxyandrost-4-en-3-one (testosterone; T), 17α-hydroxyandrost-4-en-3-one (epitestosterone; E), 3α-hydroxy-5α-androstan-17-one (androsterone; And), 5α-androstane-3,17-dione (5α-androstanedione; 5αAD), 17β-hydroxy-5α-androstan-3-one (5α-dihydrotestosterone; 5αDHT), 5α-androstane-3α,17β-diol (5αAdiol), 3α-hydroxy-5β-androstan-17-one (etiocholanolone; Etio), 5β-androstane-3,17-dione (5β-androstanedione; 5βAD), 17βhydroxy-5β-androstan-3-one (5β-dihydrotestosterone; 5βDHT), 5β-androstane-3α,17β-diol (5βAdiol) and 17α-methyltestosterone (MeT; used as internal standard) were from Steraloids (Newport, RI, USA), Sigma-Aldrich (Milano, Italy) or TCI (Eschborn, Germany). Deuterated standards T-d3, E-d3, And-glucuronide-d4, Etio-d5, 5αAdiol-d3, 5βAdiol-d5 were obtained from the National Measurement Institute (NMI, Lindfield, NSW, Australia). Indomethacin, *O*-desmethylindomethacin and *N*-deschlorobenzoylindomethacin used as reference compounds for the excretion study were all purchased from TRC (Toronto Research Chemicals, North York, ON, Canada). Probenecid as internal standard was from Sigma Aldrich (Milan, Italy). Indomethacin, used as inhibitor for in vitro experiments, was of European Pharmacopoeia (Ph. Eur.) quality and purchased from Sigma-Aldrich (Steinheim, Germany). For the in vitro assay, solutions of different concentrations of the substrate Etio (20 µM, 100 µM, 200 µM, 400 µM, 1 mM, 2 mM) and the inhibitor indomethacin (6 µM, 20 µM, 60 µM, 200 µM, 600 µM, 2 mM) were prepared from stock solution by dilution of the solid substance in DMSO in the case of Etio and in ethanol in the case of indomethacin. Solvents (ethanol, acetonitrile, DMSO, tert-butyl methyl ether (TBME), methanol or ethyl acetate) and reagents (sodium phosphate and sodium hydrogen phosphate, potassium carbonate and sodium hydrogen carbonate, acetic acid and sodium acetate) were of analytical, high performance liquid chromatography (HPLC) or of high performance liquid chromatography-mass spectrometry (HPLC-MS) reagent grade and were purchased from Merck (Darmstadt, Germany), Carl Roth (Karlsruhe, Germany), Honeywell Fluka (Milan, Italy) or Sigma Aldrich (Milan, Italy). NADPH regenerating system solution A and solution B were used for the overview incubations and were purchased from Corning Gentest (Woburn, MA, USA). The cofactor for the enzymatic incubations to determine the kinetic values K_m_ and IC_50_ was NADPH-tetra-sodium salt (NADPH-Na_4_) and was purchased from Carl Roth (Karlsruhe, Germany). The human recombinant enzyme AKR1C3 (expressed in *Escherichia coli* (*E. coli*, catalogue number: NBC1-21051) originated from Novus Biologicals Europe (Abingdon, United Kingdom). Phosphate buffered saline (PBS, 0.5 M, pH 7.4) from Corning Gentest (Woburn MA, USA) was diluted and used for all in vitro experiments. The preparation of β-glucuronidase from *E. coli* was from Roche Diagnostic (Mannheim, Germany). *N*-methyl-*N*-(trimethylsilyl)trifluoroacetamide (MSTFA) was supplied by Chemische Fabrik Karl Bucher GmbH (Waldstetten, Germany), ammonium iodide (NH_4_I), mercaptoethanol and ethanthiol were from Sigma Aldrich (Milano, Italy or Taufkirchen, Germany). Water was obtained from a “MilliQ” (Millipore S.p.A., Milano, Italy) or from “SG LaboStar” (Guenzburg, Germany) water purification system.

### 4.2. In Vitro Qualitative Incubation

Incubations were performed in duplicate in parallel to a background sample where the volume of enzyme was substituted by PBS 0.1 M. NADPH regenerating system (consisting of 1.3 mM NADP^+^, 3.3 mM glucose-6-phosphate and 3.3 mM magnesium chloride and 0.4 U/mL glucose-6-phosphate dehydrogenase in final assay), AKR1C3 (0.64 µM in final assay) and PBS 0.1 M were added to a 1.5 mL reaction tube, centrifuged briefly and prewarmed at 37 °C for 5 min. AED, And, 5αAD, 5βAD or Etio (dissolved in methanol or acetonitrile; concentration in stock solution 1 mg/mL) were added as substrates (final concentration in assay: steroid ~50 µM, solvent 1.5% (*v*/*v*)). After brief centrifugation, incubation was carried out for 5.5 h at 37 °C. Incubation was terminated by the addition of 200 µL cold acetonitrile and samples were extracted immediately or stored at −20 °C until extraction.

### 4.3. In Vitro Kinetic Assay

All incubations were performed in triplicate. The incubations were carried out in 96-well plates (black, flat black bottom, Sarstedt, Nümbrecht, Germany) and analyzed spectro-fluorometrically in real-time. After incubation, the entire assay-volume was transferred to a 0.5 mL reaction tube and 200 µL of cold acetonitrile were added to denature the enzyme and thereby end the reaction. Samples were stored at −20 °C until confirmative GC-QToF analysis was performed. For each experiment NADPH solution (in PBS 0.1 M) was prepared freshly. Ethanol or indomethacin dissolved in ethanol, NADPH (12 µM in final assay) in PBS 0.1 M, AKR 1C3 (1.03 µM in final assay) and PBS 0.1 M were added to the appropriate location on the well-plate. The mixture was prewarmed for 3 min at 37 °C under agitation. After the addition of Etio, the plate was centrifuged briefly, and incubation was performed for 1000 s in the plate reader. Concentrations of Etio for Michaelis-Menten-constant (K_m_)-determination were 1 µM, 5 µM, 10 µM, 20 µM, 50 µM or 100 µM in the final assay. In experiments to evaluate the half maximal inhibitory concentration (IC_50_), Etio was used in a concentration slightly below the determined K_m_ (7 µM in final assay) throughout. Indomethacin (dissolved in ethanol) was added, resulting in concentrations of 0.1 µM, 0.3 µM, 1 µM, 3 µM, 10 µM, 30 µM or 100 µM in final assay.

### 4.4. Administration Study

Two healthy volunteers, one male and one female (additional information on volunteers in Table 1), were treated with indomethacin (Indoxen 25 mg; Recordati S.p.A.; Milan, Italy) for 14 days. Urine samples were collected 4 times per day for five days prior to the treatment and the whole treatment phase (sampling points: 07:00 h; 13:00 h, 18:00 h and 22:00 h) and one time per day after the treatment phase (sampling point 13:00 h over 11 days for male volunteer and 8 days for female volunteer). Urine samples were anonymized and stored in sterile plastic tubes at −20 °C until analysis. The study was approved by the “Comitato Etico Lazio 1” with the reference number: 1553/CE Lazio 1. All volunteers gave informed consent.

### 4.5. Sample Preparation before Chromatographic Analysis

In case of in vitro samples (overview incubations and kinetic experiments), MeT (in methanol; 10 µL of 1 g/L-solution for overview study or 10 µL of 0.1 g/L-solution for kinetic study) was added. Samples were centrifuged (at 9660× *g*) to sediment the denatured enzyme. Supernatant was transferred to fresh glass-tubes and 1 mL PBS 0.1 M and 5 mL TBME (in case of kinetic experiments) or 2 mL ethyl acetate (in case of the overview incubations) were added.

For all urine samples, specific gravity was determined, to normalize steroid and NSAID concentrations according to the WADA technical document TD2018EAAS, using a digital refractometer RM40 (Mettler Toledo, Novate Milanese, Italy) [3]. Steroids (free and glucuronide fraction) were extracted using an already established protocol for steroid profiling of endogenous steroids, which is used routinely in the Italian anti-doping laboratory, Rome [34,35,36]. In brief, 2 mL of urine, 30 μL of β-glucuronidase, 750 μL of 0.8 M PBS and 50 μL of internal standard (T-d3: 100 ng/mL, E-d3: 25 ng/mL, And-d4 glucuronide: 200 ng/mL, Etio-d5: 200 ng/mL, 5αAdiol-d3: 50 ng/mL, 5βAdiol-d5: 50 ng/mL, MeT: 250 ng/mL) were mixed and heated for 1 h at 55 °C. After hydrolysis, 500 μL of carbonate/bicarbonate buffer (20% (*w*/*v*), pH 9) and 5 mL TBME were added.

For the extraction of indomethacin and its metabolites, 1 mL of urine, 50 µL probenecid (1 µg/mL) as internal standard, 30 µL of β-glucuronidase and 500 µL PBS 0.8 M were added and hydrolysis was achieved after 1 h of incubation at 55 °C. Before extraction, 500 µL sodium acetate buffer (pH 5) and 5 mL TBME were added.

For in vitro and urine samples, liquid-liquid extraction was performed during agitation on an automated shaker. Samples were centrifuged briefly (relative centrifugal force was 1008× *g*) to separate the phases. The organic layer was transferred to new glass tubes after 5 min freezing in an ethanol bath (−25 °C) or overnight at −20 °C in the freezer. The solvent was evaporated under nitrogen at elevated temperatures and derivatization was performed at 75 °C for 30 min with 50 µL MSTFA/mercaptoethanol/NH_4_I (1000:6:4 *v*/*v*/*w*; in case of in vitro kinetic samples and urine samples) or MSTFA/ethanthiol/NH_4_I (1000:3:2 *v*/*v*/*w*; for overview incubations).

### 4.6. Fluorometric Analysis

Spectro-fluorometric measurements to determine the kinetic values of the enzymatic reaction were carried out on a Safire II microplate reader (Tecan, Männedorf, Switzerland) in top-read mode. Excitation wavelength was 340 nm, emission wavelength was 450 nm with both bandwidths set to 5 nm. For all experiments, gain was 120 and Z-position was 10,300 µm. Time between move and flash was 10 ms, integration time 40 µs and 0 µs lag time. In the beginning of all experiments performed on the plate reader, a calibration with 8 different concentrations of NADPH in PBS 0.1 M was performed. For each concentration, 200 µL of NADPH solution were pipetted to 10 individual wells and each well was measured with the same method used for kinetic experiments. The resulting calibration curve was tested for linearity (Mandel-test, *p* = 99%), homogeneity of variances following DIN 38 402 A51 (*p* = 99%, including Grubbs outlier-test) and limit of quantification (LOQ) was determined following DIN 32 645 (*p* = 95%, confidence level: 95%).

### 4.7. GC-MS Analysis

Samples originating from overview incubations were analyzed on an Agilent 7890A GC-System coupled to an Agilent 5975C MSD (Agilent Technologies, Santa Clara, CA, USA). Injection volume was 2 µL with split injection in a ratio of 1:10. Injection temperature was set to 300 °C. Chromatographic separation was achieved on an Agilent HP1 column (length: 17 m; diameter: 0.2 mm; film-thickness: 0.11 µm) with helium as carrier gas and a flow-rate of 1 mL/min. Oven program started at 193 °C with 3 °C/min heating rate to 215 °C followed by a ramp with 40 °C/min to 310 °C and hold for 1 min. Ionization was electron ionization (EI) and analysis was performed in scan mode with acquisition from *m/z* 40 to *m*/*z* 750.

### 4.8. GC-QToF Analysis

Instrumental analysis of kinetic in vitro samples was performed on an Agilent GC-QToF 7890B/7200 (Agilent Technologies, Milano, Italy), equipped with an Agilent HP1 column (length: 17 m; diameter: 0.2 mm; film-thickness: 0.11 µm) with helium as carrier gas and a flow of 0.8 mL/min. Injection was performed in split mode with a 1:10 ratio at 280 °C. The oven program had the following heating rates: 188 °C hold for 2.5 min, 3 °C/min to 211 °C and hold for 2 min, 10 °C/min to 238 °C, 40 °C/min to 320 °C and hold for 3.2 min. The coupled QToF was operated in full scan with an EI source and ionization energy of 70 eV. Ions were detected from *m/z* 50 to *m/z* 750.

Calibration was performed with 9 different concentrations of 5βAdiol with fixed concentrations of MeT. Each calibration level was measured twice. Calibration was tested for linearity applying the Mandel Test, tested for outliers (no residuals >3 standard deviations) and weighted with a factor 1/x. A signal to noise ratio of 10 to 1 was regarded as LOQ. Samples with signals below the LOQ were regarded as not containing any analyte.

### 4.9. GC-MS/MS Analysis

For urinary steroid profile analyses, a previously described method was used [34,35,36]. It is validated and currently used in routine analysis for the detection and quantification of pseudo-endogenous steroids in the Italian anti-doping laboratory in Rome and accredited under ISO17025. In brief, analyses were performed on an Agilent GC-MS/MS 7890 A/7000 (Agilent Technologies, Milano, Italy) equipped with an Agilent HP1 column (length: 17 m; diameter: 0.2 mm; film-thickness: 0.11 µm). The carrier gas was helium with a flow rate of 1 mL/min, and the injection and transfer line temperature were set to 280 °C and injection was performed in split mode with a ratio of 1:20. The oven program was as follows: 180 °C hold for 4.5 min, 3 °C/min to 230 °C, 20 °C/min to 290 °C and 30 °C/min to 320 °C. Ionization was achieved by EI and ionization energy was 70 eV. Analyses were performed in multiple-reaction monitoring mode (MRM mode) with transitions used in the reference method. Quantitation of the urinary steroids was based on the peak area ratio of the analyte to the corresponding internal standard. Calibration and quality control samples were prepared in synthetic urine according to previously published methods [37].

For quantification of indomethacin and its metabolites, a similar method was used. Aberrantly, the oven program was 85 °C initial temperature hold for 1 min, 15 °C/min to 320 °C. Transitions for analyzed compounds are listed in Table 2. Selectivity, linearity, repeatability, recovery and limit of detection (LOD) were tested for the evaluation of method suitability (data not shown).

### 4.10. Data Analysis of Kinetic Values

To determine the Michalis-Menten constant, initial velocities of the enzymatic reactions were plotted against the concentration of Etio. Nonlinear regression was applied to the data using OriginPro, Version 2019 (OriginLab Corporation, Northhampton, MA, USA). For spectro-fluorometric measurements, the loss in fluorescence intensity over time curves were transformed in loss of NADPH-concentration over time using a calibration curve. By applying linear regression to those concentration-time curves, the initial velocity was determined. Initial velocities for the individual samples were corrected with the background velocities. For mass spectrometric data the determined concentration of formed product (5βAdiol) over time of incubation was regarded as initial velocity. As no product was present in background incubations, no correction of this value was performed. GC-QToF data were analyzed using Agilent MassHunter Workstation Quantitative Analysis for TOF (Version 10.1; Agilent Technologies, Santa Clara, CA, USA).

To determine the IC_50_ values of the inhibition experiments, activity of the enzyme was plotted against logarithmic concentrations of the inhibitor indomethacin in the assay. The activity of the enzyme was calculated as a quotient of the initial velocities of the sample and the initial velocities of the positive control (no inhibitor added). Nonlinear regression was performed using OriginPro, Version 2019.

### 4.11. Data Analysis of Steroid Profiling and Indomethacin Detection in Urine Samples

Measured concentrations of EAAS, indomethacin and its metabolites were normalized for the specific gravity of the sample according to the WADA technical document TD2018EAAS [3]. Samples with a specific gravity at or below 1.001 were excluded from the analysis. For samples in which selected analytes had concentrations below the limit of quantification (LOQ), the analyte in question and its related ratios were excluded from analysis.

For the steroid-profiling samples of each volunteer were divided into three groups: before treatment with indomethacin (*n* = 20) during the administration (*n* = 56 for male volunteer; *n* = 55 for female volunteer due to exclusion of one sample where specific gravity was 1.001 and *n* = 54 for female volunteer for E, T/E, 5αAdiol/E because concentration of E was below LOQ in this sample) and after indomethacin intake (*n* = 11 for male volunteer and *n* = 8 for female volunteer). The data were regarded as independent from each other. All measured concentrations belonging to one group (per volunteer, per substance, and phase during treatment) were tested for normal distribution (Shapiro Wilk test, α = 0.05). In case of normal distribution the homogeneity of variances between the groups was also tested (F-Test, α = 0.05). If the hypothesis of normal distribution was not rejected for both groups examined, a two-sided parametric significance test for independent samples was applied. In case of homogeneity of variances between groups, a two-sided t-test for independent groups was applied. In case of rejection of homogeneity of variances between groups, Welch-Test was used. When for at least one of the two examined groups the null-hypothesis of normal distribution had to be rejected, the less powerful non-parametric Mann-Whitney U-Test was applied. For all significance tests (parametric and non-parametric) the significance level was α = 0.0167 to deal with multiplicity problems of statistical tests [38].

Normalized concentrations of indomethacin and its metabolites were plotted against the time after first dose of indomethacin was administered.

## 5. Conclusions

The presented work shows the complexity of steroid metabolism in vitro and in vivo. It furthermore reveals the advantages and drawbacks of different analysis methods for in vitro enzymatic assays. A combination of in vitro and in vivo experiments was performed showing the interference of the non-steroidal anti-inflammatory drug indomethacin with androgen metabolism and the effect of indomethacin on the urinary steroid profile. The study provides results which can help to interpret steroid profiles in anti-doping analysis. The presented results may also be relevant for other fields of forensic or clinical toxicology and for the investigation of endocrine disrupting substances (EDS). Future investigations on long-term effects or the influence of higher doses of indomethacin may extend our findings.

## Figures and Tables

**Table 1:** Additional information on male and female volunteer included in this study.

	Male Volunteer	Female Volunteer
Age	29	30
Weight	63 kg	60 kg
Hight	172 cm	178 cm
BMI	21.3 kg/m^2^	18.9 kg/m^2^
Dietary habits	Normal diet (no restriction)	Normal diet (no restriction)
Training	Three times/week (swim and run)	No
Oral contraceptive	Ø	No

**Table 2:** Transitions for GC-MS/MS analysis of indomethacin and its metabolites *O*-desmethylindomethacin and *N*-deschlorobenzoylindomethacin and the internal standard probenecid.

Analyte	Precursor Ion [*m/z*]	Product Ion [*m/z*]	Collision Energy [eV]
Indomethacin (mono-TMS)	429	139; 246; 312	30; 30; 30
*O*-desmethylindomethacin (bis-TMS)	487; 304	139; 216; 232	30; 30; 30
*N*-deschlorobenzoylindomethacin (bis-TMS)	363; 348; 246	246; 320; 174	30; 10; 30
Probenecid (mono-TMS)	178; 342	104; 268	20; 20

## Supplementary Materials

The following are available online at https://www.mdpi.com/2218-1989/10/11/463/s1. Supplement S1: Chromatograms of in vitro qualitative incubation.
