# Supplementary material for: Influence of Indomethacin on Steroid Metabolism: Endocrine Disruption and Confounding Effects in Urinary Steroid Profiling of Anti-Doping Analyses
Source: Metabolites. 2020 Nov 14;10(11):463. doi: 10.3390/metabo10110463 (PMC7698016; doi:10.3390/metabo10110463)

# "Influence of indomethacin on steroid metabolism: Endocrine disruption and confounding effects in urinary steroid profiling of anti-doping analyses"

## Supplement S1: Chromatograms of in vitro qualitative incubation

**Anna Stoll [1], Michele Iannone [2], Giuseppina De Gregorio [2], Francesco Molaioni [2], Xavier de la Torre [2], Francesco Botrè [2,3] and Maria Kristina Parr [1,*]**

1 Freie Universität Berlin, Institute of Pharmacy (Pharmaceutical and Medical Chemistry), 14195 Berlin, Germany; anna.stoll@fu-berlin.de (A.S.); maria.parr@fu-berlin.de (M.K.P.)

2 Laboratorio Antidoping Federazione Medico Sportiva Italiana, 00197 Rome, Italy; micheleiannone14@gmail.com (M.I.); degregorio.giuseppina@gmail.com (G.D.G.); molaioni@gmail.com (F.M.); xavier.delatorre@gmail.com (X.T.)

3 ISSUL – Institute del sciences du sport, Université de Lausanne, Synathlon – Quartier Centre, 1015 Lausanne, Switzerland; Francesco.Botre@unil.ch (F.B.)

* Correspondence: maria.parr@fu-berlin.de; Tel.: +49-30-838-51471

The following document displays chromatograms obtained after incubation of the endogenous anabolic androgenic steroids (EAAS) 5α-androstanedione, 5β-androstanedione, androstenedione, androsterone and etiocholanolone with isolated recombinant human aldo-keto reductase (AKR) 1C3.

Similarely to the main document all 5α-androstanes are highlighted in organge, all 5β-androstanes are highlighted in green, testosterone and androstenedione are highlighted in violett and the internal standard methyltestosterone (MeT) is highlighted in grey.

To simplify the graphics, substrates are indigated with framed retention times (RT) and all detected EAAS are labeled with abbreviations: A: androsterone, 5αAdiol: 5α-androstanediol, 5αAD: 5α-androstanedione, 5αDHT: 5α-dihydrotestosterone, Etio: etiocholanolone, 5βAdiol: 5β-androstanediol, 5βAD: 5β-androstanedione, 5βDHT: 5β-dihydrotestosterone, T: testosterone, AED: androstenedione.

Comment: As the peak corresponding to 5βDHT in sample 1 (incubation of 5βAD with AKR1C3) is very small, a zoomed segment of the chomatogram is provided.

# 5αAndrostanedione

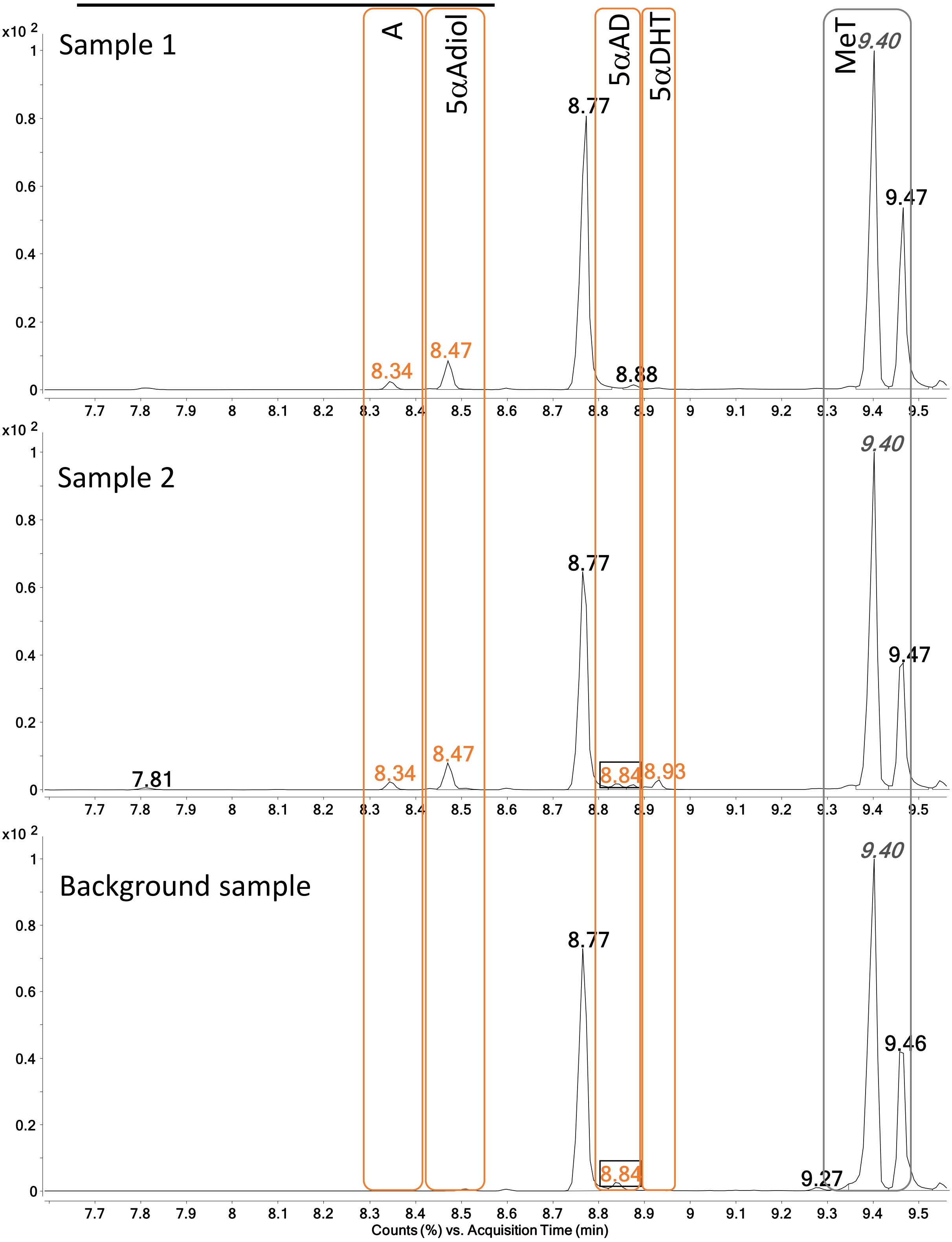

# 5βAndrostanedione

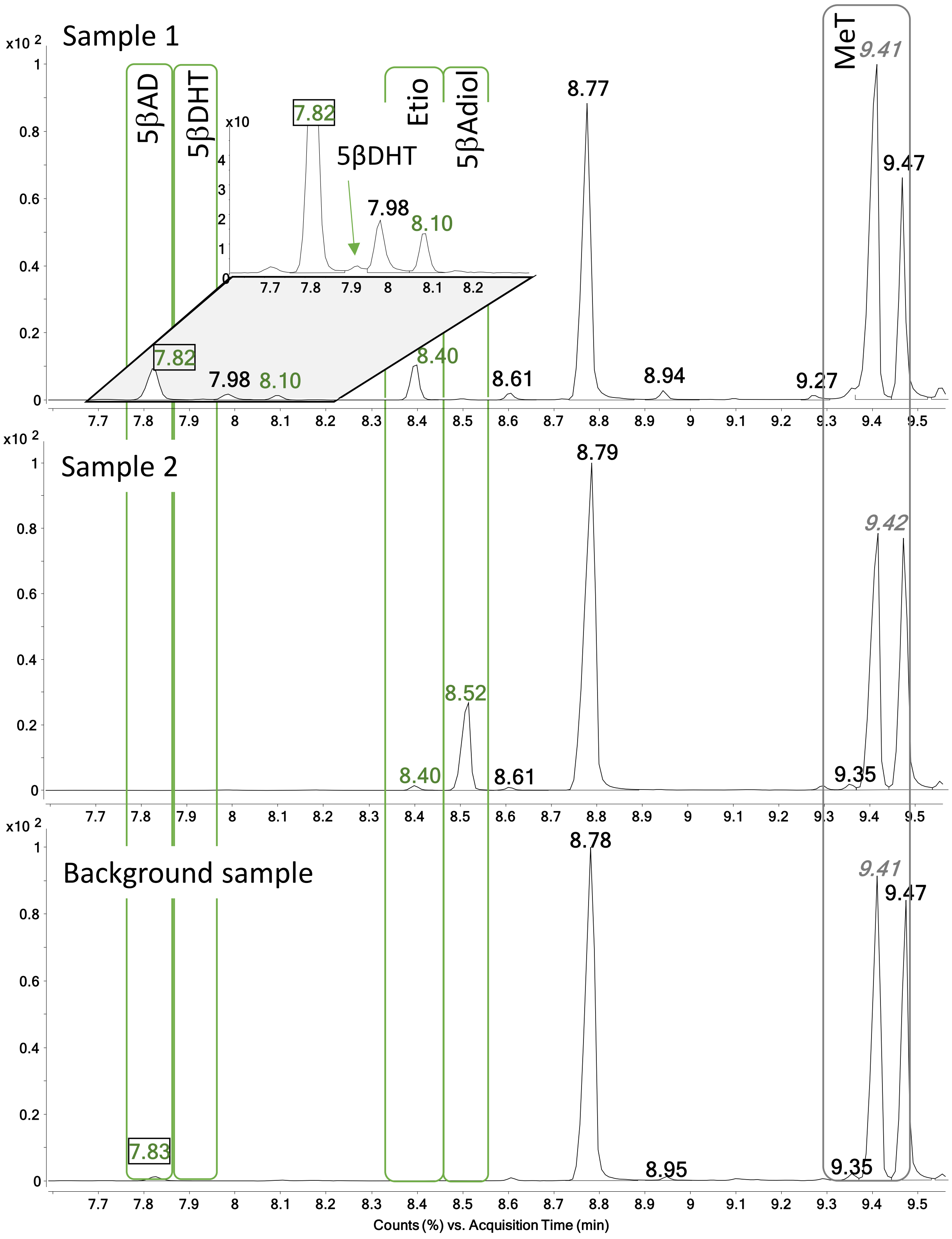

# Androstenedione

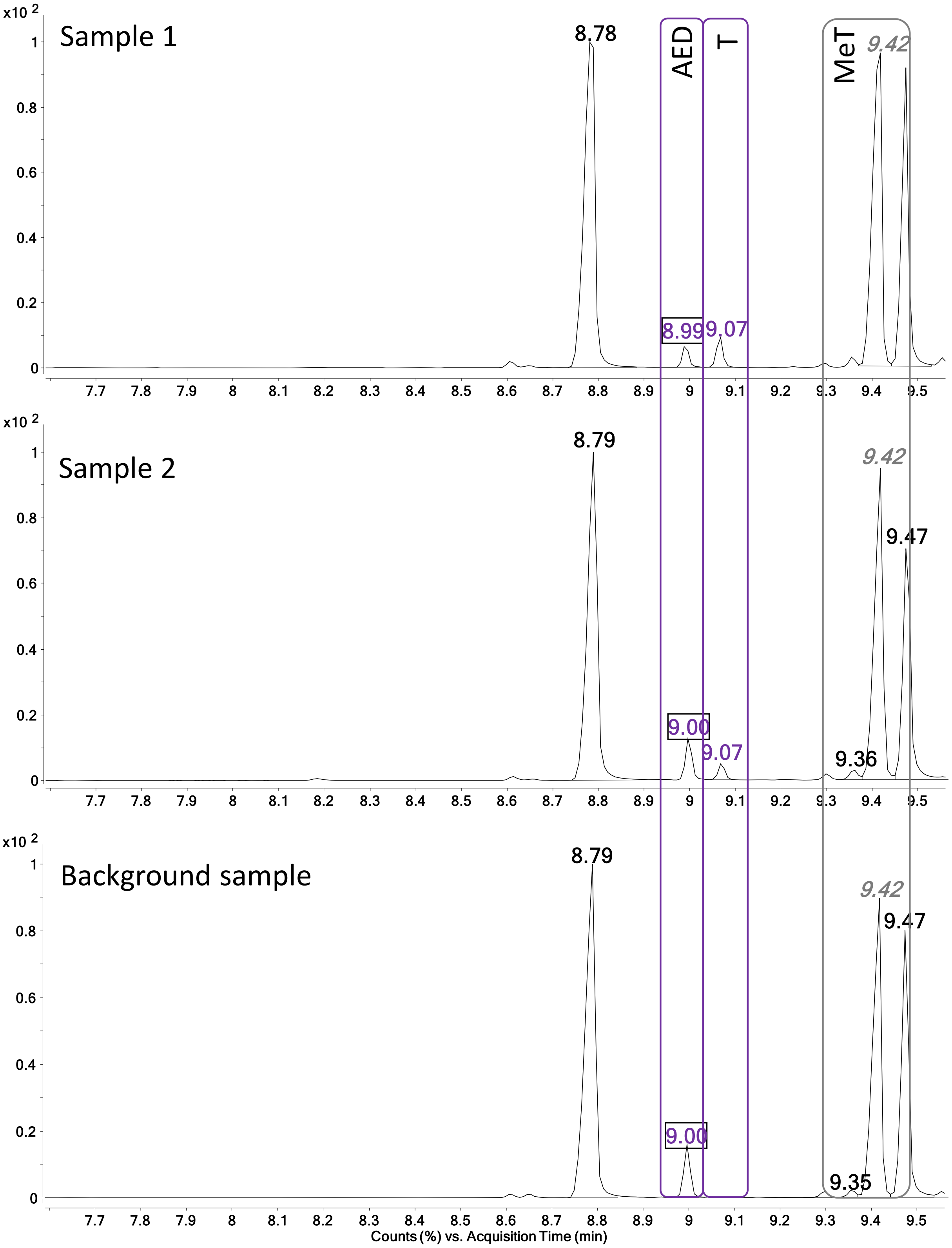

# Androsterone

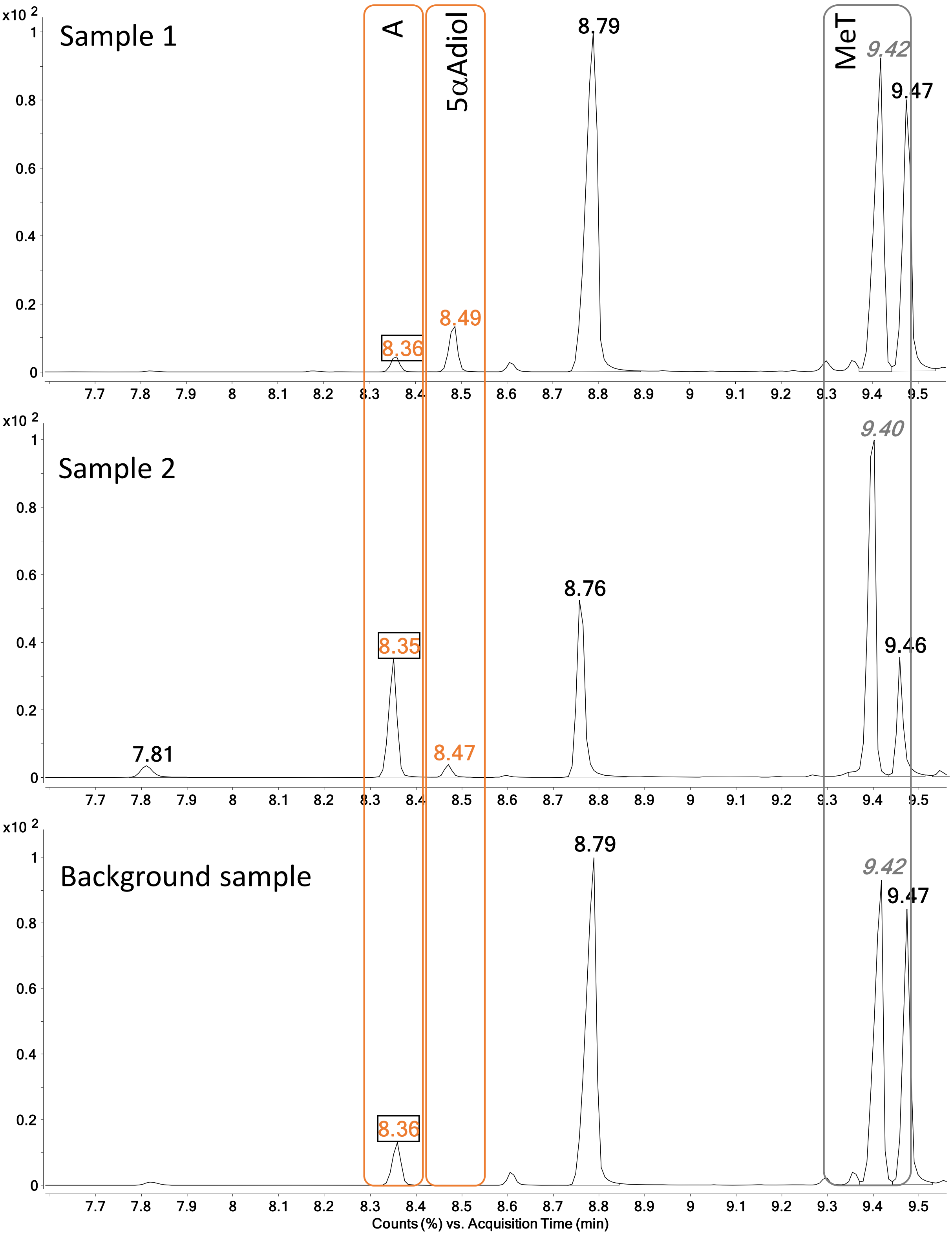

# Etiocholanolone

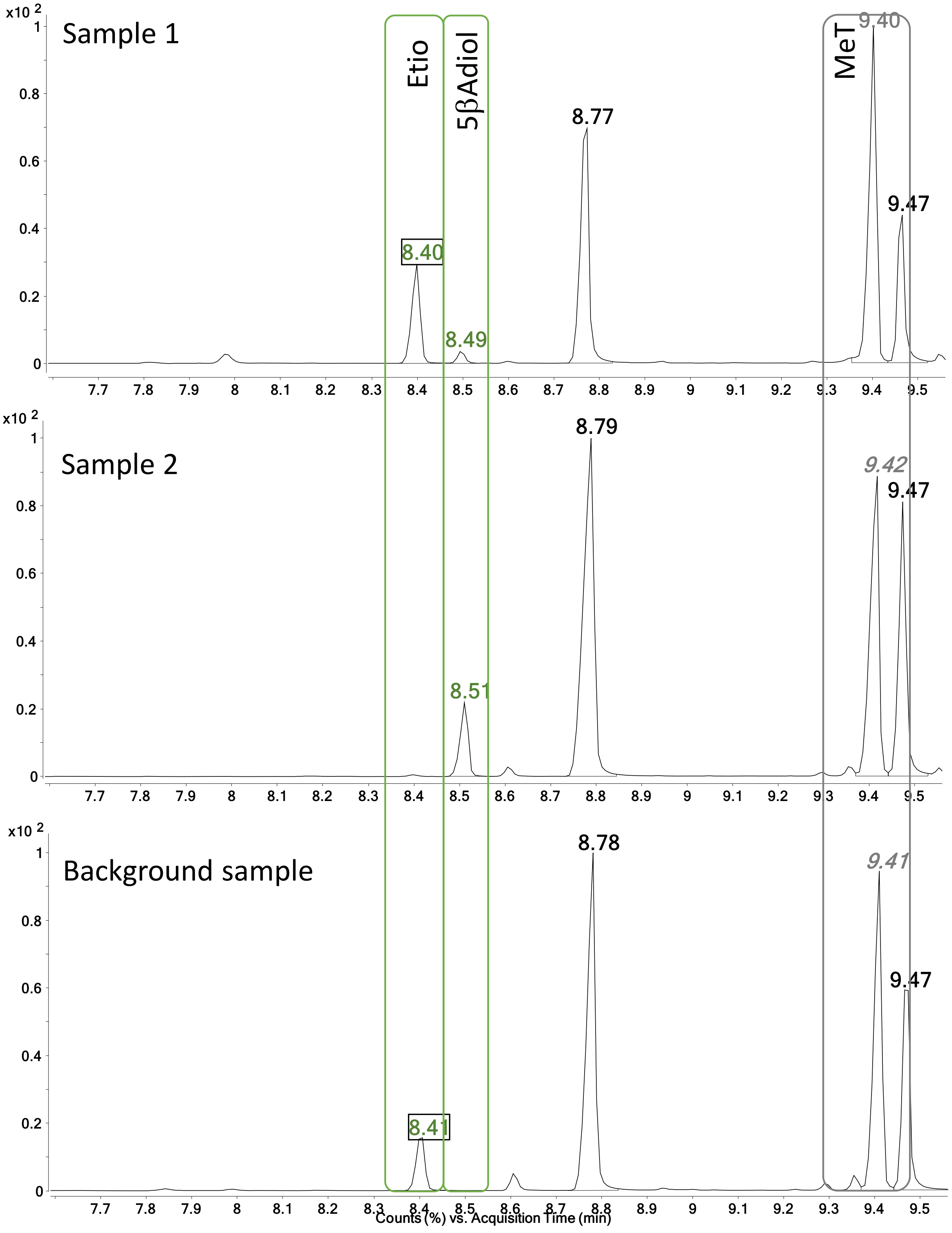

Supplement: Supplementary file 1 [file metabolites-10-00463-s001.pdf]
